# Epidemiological Trends of Diabetes Mellitus and Human Immunodeficiency Syndrome and Their Effect on Treatment Outcomes in Patients With Tuberculosis: A Situational Analysis of Tuberculosis Elimination Program Data From Tirunelveli District, 2017–2021

**DOI:** 10.7759/cureus.79792

**Published:** 2025-02-27

**Authors:** Prateeksha D Davidson, Sudharsan Vasudevan, Aishwariya Radhakrishnan, Vellasamy S, Sethu Madhavan

**Affiliations:** 1 Biostatistics, Indian Council of Medical Research (ICMR) Model Rural Health Research Unit (MRHRU), Chennai, IND; 2 Public Health, Indian Council of Medical Research (Model Rural Health Research Unit), Tirunelveli, IND; 3 Epidemiology and Public Health, Public Health Foundation of India (PHFI), Kochi, IND; 4 Program Managment, Directorate of Public Health and Preventive Medicine, Chennai, IND; 5 Program Coordination, National Tuberculosis Elimination Program (NTEP), Tirunelveli, IND

**Keywords:** comorbidities of tuberculosis, diabetes and tb, hiv and tb, ntep, tuberculosis

## Abstract

Background

Tuberculosis (TB) was the leading cause of death due to a communicable disease before the COVID-19 pandemic. In 2021, India accounted for 25% of the world’s TB cases. Tamil Nadu has a higher TB prevalence than the national average. Individuals with diabetes mellitus (DM) are two to four times more likely to develop active TB, while people living with human immune deficiency syndrome (PLHIV) have 20 times greater odds. This study aims to describe the epidemiological trends of comorbidities in TB cases over time and assess the relationship between comorbidities and treatment outcomes.

Methods

A secondary data analysis of NIKSHAY data was conducted for the Tirunelveli district from 2017 to 2021.

Results

The proportion of TB patients with DM increased significantly from 3.5% in 2017 to 27% in 2021. TB units in Pettai (25%) and Vadakkankulam (18%) had the highest percentage of patients with DM. HIV prevalence among TB patients was highest in Manur (2.2%), followed by Vadakkankulam (2.1%) and Papakudi (1.9%). Patients with DM had 33% higher odds of mortality compared to those without DM, while TB patients with HIV were 87% more likely to die from the disease (p < 0.000).

Conclusion

This study highlights the evolving trends of DM and PLHIV among TB patients and their impact on treatment outcomes in the Tirunelveli district. The effect of these comorbidities on mortality has been reiterated in this study.

## Introduction

Tuberculosis (TB) a leading infectious disease is a global public health issue, with over 10 million new cases and 1.13 million deaths reported in 2022 alone, according to the World Health Organization (WHO) [1,2]. India continues to carry a disproportionate burden of TB, accounting for approximately 25% of the global incidence [1]. The nation's TB control efforts are primarily guided by the National Tuberculosis Elimination Program (NTEP), which works to eliminate TB by 2025, ahead of the global targets [3,4]. Tamil Nadu is a large state in the country, which has a large aging population, because of which a higher proportion of lifestyle illnesses. The increasing prevalence of comorbidities such as diabetes mellitus (DM) and HIV among TB patients is posing new challenges to TB control programs, particularly in regions like Tirunelveli district in Tamil Nadu, which is the district the study is based in.

Epidemiological trends of comorbidities

The coexistence of DM and TB has been well-documented and is recognized as a critical factor in the progression of both diseases. In India, the prevalence of DM is rising rapidly, with projections estimating between 62 to 80 million people living with diabetes by 2030 [5-7]. Diabetes, known to impair immune function and disrupt granuloma formation, significantly increases susceptibility to active TB [5-7]. It is reported that 13% of TB patients in India had DM, with a substantial proportion of these individuals being newly diagnosed during their TB treatment. This comorbidity complicates the management of TB, as it is associated with increased risk of treatment failure, relapse, and mortality [6-8].

Further exacerbating the burden of TB, the presence of HIV infection in TB patients increases the likelihood of poor outcomes. Individuals living with HIV (PLHIV) are 20 times more likely to develop active TB compared to those without HIV, and TB remains a leading cause of death among PLHIV [9]. The coexistence of HIV with TB leads to an increased risk of mortality, delayed diagnosis, and complications in TB treatment [8,9].

The impact on treatment outcomes

The presence of both DM and HIV among TB patients is linked to worse treatment outcomes, including higher mortality rates, and delayed treatment responses. A growing body of evidence indicates that TB patients with DM face significantly higher odds of death compared to those without diabetes [7,8]. Similarly, HIV-infected TB patients experience elevated mortality, as immune suppression due to HIV hampers the body’s ability to fight TB infection [8-10]. Moreover, these comorbidities increase the risk of multidrug-resistant TB (MDR-TB), further complicating treatment protocols. Studies have demonstrated that patients with DM are more likely to develop MDR-TB due to poor medication adherence and impaired immune function [9-11].

The prevalence of these comorbidities is notably rising in areas like Tirunelveli, where the increasing incidence of DM and HIV among TB patients is becoming a critical challenge for health authorities [12]. This necessitates the need for integrated care models that simultaneously address TB, DM, and HIV to improve treatment outcomes and reduce the burden of these coexisting epidemics.

Programmatic management of comorbidities

The rise of these comorbidities across different regions of India, including Tirunelveli, highlights the importance of region-specific interventions. The NTEP's focus on eliminating TB, has also successfully integrated diabetes and HIV screening into routine TB care as a critical step towards improving early diagnosis and initiating appropriate treatment. Such integrated approaches have been shown to improve treatment adherence, reduce mortality, and lower the incidence of drug resistance [11,13]. The need for enhanced surveillance systems, better coordination between TB, diabetes, and HIV control programs, and region-specific strategies is needed in districts like Tirunelveli, where the dual burden of communicable and non-communicable diseases significantly impacts TB treatment outcomes.

The study aims to understand the epidemiological trends of these comorbidities in Tirunelveli and its blocks, programmatic efficiency in diagnosing these comorbidities among TB cases, and find their effect on treatment outcomes like mortality, treatment failure and drug resistance.

## Materials and methods

Study design

This study is a retrospective secondary data analysis of NTEP data collected in Tirunelveli district between 2017 and 2021.

Study population

The study included 13,077 TB patients from the NIKSHAY portal in Tirunelveli district. This data was obtained with the permission of the NTEP program in Tirunelveli for developing the situational analysis of the program in the district.

Data sources

NTEP-related data were obtained from the NIKSHAY portal. Demographic information was collected from the public health department in Tirunelveli district.

Statistical analysis

Trends in TB notifications with and without DM and HIV were analyzed over time across the 12 TB units. Descriptive statistics like percentages were used for representation of TB cases. Regression analysis was performed to estimate the effect of comorbidities on treatment outcomes and drug resistance. Graphs were created using Microsoft Excel (Redmond, WA, USA), and epidemiological maps were generated using Quantum Geographic Information System version 3.38.0 RC, an incubator project of Open Source Geospatial Foundation (Beaverton, OR, USA).

## Results

The highest average TB notifications over the five-year period were observed in the TB units of Samadanapuram (6,664), Melaveeraragavapuram (1,599), and Palayamkottai (878), all of which are urban localities within the Tirunelveli Municipal Corporation. The TB unit covering the largest population is Radhapuram (220,000), according to the mid-year population of 2021, with an average of 210 TB notifications from this unit. The unit with the smallest population is Kalkadu, which recorded a cumulative TB notification of 177. The predominant number of TB patients fell within the age range of 45-60 years, with the largest proportion of elderly patients (>60) located in the TB units of Melaveeraragavapuram, Palayamkottai, and Samadanapuram. In 2021, 27 individuals (93%) diagnosed with HIV were started on antiretroviral therapy (ART), showing significant improvement from 2017, when only two individuals (7%) had documented initiation of ART.

**Figure 1:**
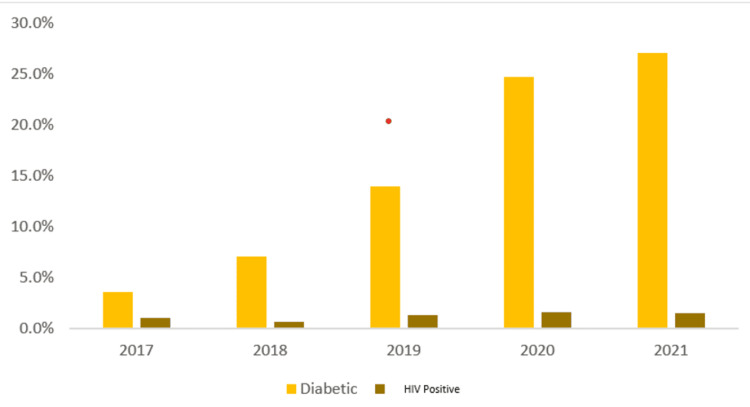
Trends of diabetes mellitus (DM) and HIV among tuberculosis (TB) patients over time (2017 to 2021). This figure describes the trend of comorbidity proportions among TB cases, the increasing trend coincides with increased testing for them. Image created by the authors.

The number of TB patients with DM steadily increased between 2017 and 2021 from 94 patients (3.5%) to 477 patients (27%) (Figure 1). The percentage of patients with unknown DM status, which had declined significantly until 2019, has since plateaued between 0.5% (n=13) and 0.4% (n=7). The number of patients who have been initiated on DM therapy has also grown steadily.

The proportion of TB cases found to have HIV has been steadily increasing since 2018, from 0.7% (n=16) to 1.5% (n=26) (Figure 1). This increase aligns with improvements in HIV testing, as more people with TB have now been tested for HIV, leading to fewer people remaining unaware of their HIV status, that is 42% (n=1142) in 2017 to 1.9% (n=33) in 2021.

The percentage of TB patients with DM is highest in the TB units of Pettai (25%, n=146), followed by Vadakkankulam (18%, n=107) and Cheranmahadevi (18%, n=114) when compared to other units. The proportion of individuals whose DM status is unknown is highest in Samadanapuram (1.9%, n=31), Kalakadu (1.7%, n=8), and Pettai (1.4%, n=8), while the rest of the units report figures below 1%.

**Figure 2:**
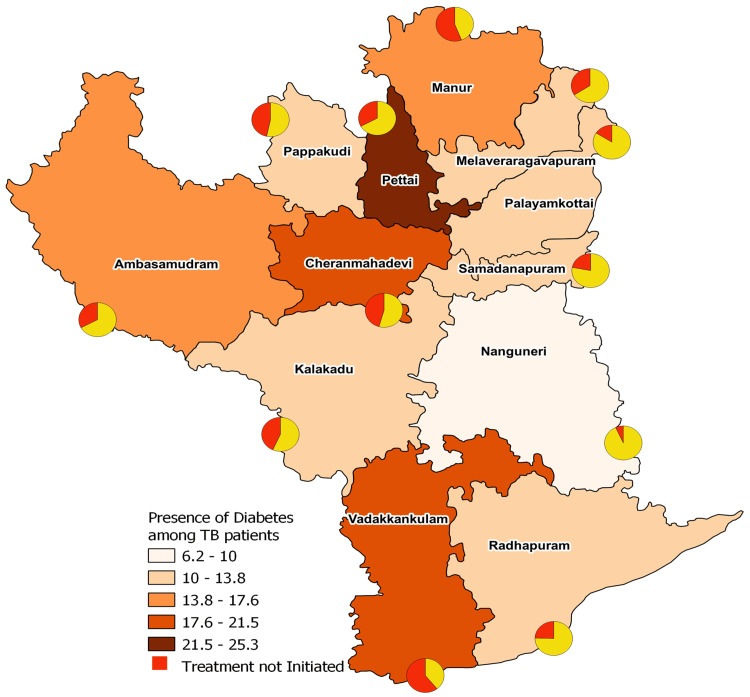
Proportion of diabetes mellitus (DM) cases among tuberculosis (TB) patients in TB units in Tirunelveli district. This figure denotes the proportions of DM cases among those notified as TB across various TB units in TIrunelveli district, Tamilnadu. The highest proportion is found in Pettai. Image created by the authors.

The percentage of patients started on anti-diabetic treatment is lowest in Vadakkankulam (39%, n=42), Manur (44%, n=49), and Papakudi (53%, n=24). TB units with high rates of anti-diabetic treatment initiation include Nanguneri (93.33%, n=28), Palayamkottai (84.06%, n=174), and Samadanapuram (78%, n=163) (Figure 2).

**Figure 3:**
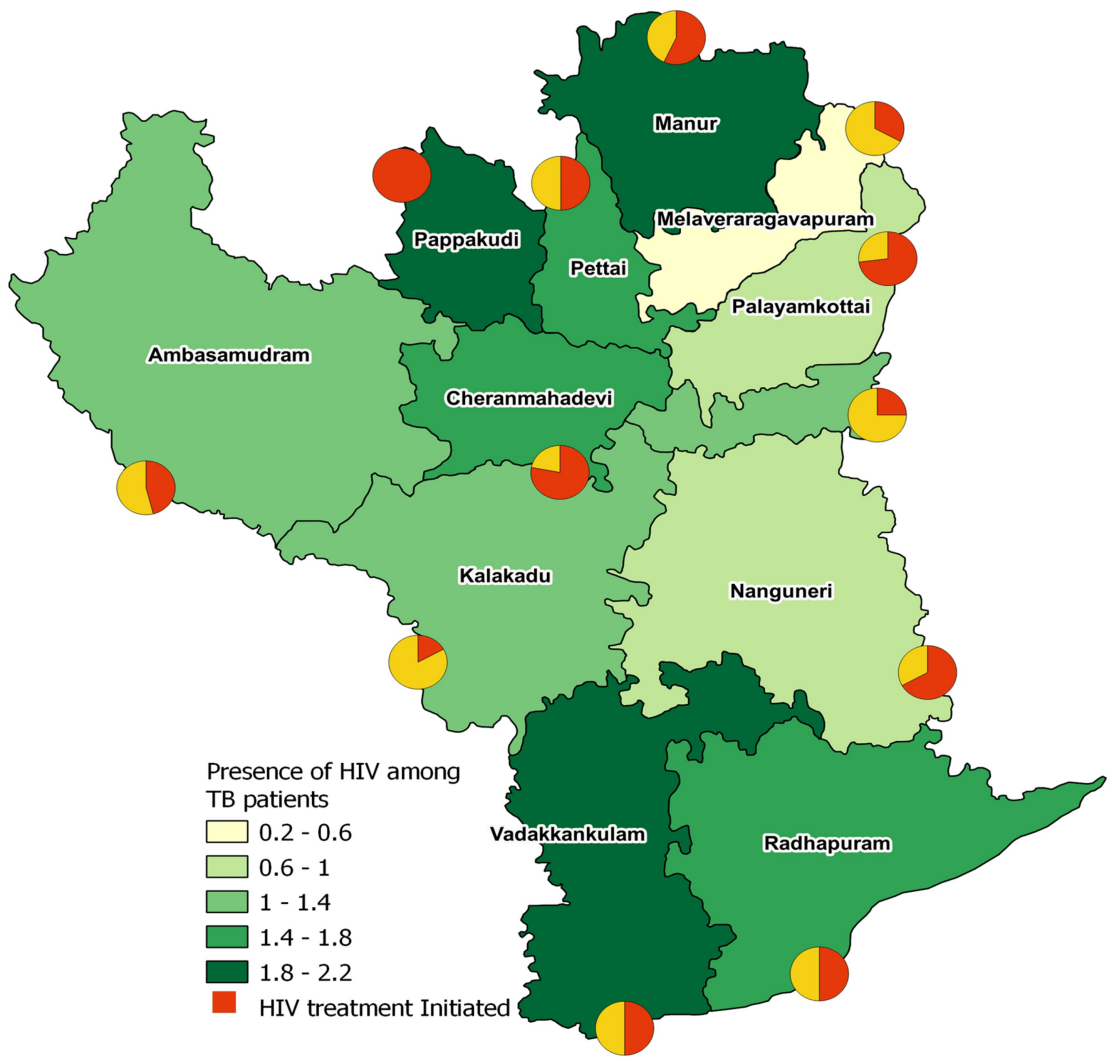
Shows the proportion of HIV cases across among tuberculosis (TB) patients, TB units in Tirunelveli district. This figure denotes the proportion of HIV cased among the TB patients across TB units in Tirunelveli district. The highest proportion of HIV cases are seen in Manur, Papakudi and Vadakkankulam. Image created by the authors.

The proportion of TB patients with HIV is highest in Manur (2.2% n=14), Vadakkankulam (2.1%, n=12), and Papakudi (1.9%, n=8). The percentage of individuals with unknown HIV status is highest in Palayamkottai (52.6%, n=924) and Melaveeraragavapuram (46.8%, n=1064). In the Papakudi TB unit, all TB patients diagnosed with HIV were initiated on treatment. Other TB units with high rates of HIV treatment initiation include Cheranmahadevi (78%, n=7) and Palayamkottai (73%, n=10). Among all TB units, Kalakadu (17%, n=1) has the lowest rate of ART initiation, followed by Samadanapuram (25%, n=6) and Melaveeraragavapuram (33%, n=2) (Figure 3).

In 2021, 93% (n=28) of those diagnosed with HIV were started on ART. This represents a significant improvement from 2017, when only 7% (n=2) of HIV-positive TB patients had initiated ART. Thirteen individuals who were not HIV-positive were erroneously documented as having been started on ART, which is likely due to human error.

The mortality rate among TB patients with HIV (13.6%, n=20) was much higher compared to the overall TB patient population (6.5%) and those with the comorbidity of DM (6.7%, n=114). Treatment failure was nonexistent among the HIV population, while it was slightly higher among TB patients with DM (2.03%, n=26) compared to the overall TB patient population (0.82%, n=85). The recurrence of TB among those treated for TB, with HIV (5.04%, n=7), was lower than those with DM (5.46%, n=92) (Table 1).

**Table 1:** Comparison of clinical outcomes between tuberculosis (TB) patients with diabetes mellitus (DM) and without DM. *Chi-square test P value ≤ 0.05: significant

		With DM		Without DM		Odds ratio (CI)	Chi-square value	*p-value
		N = 1684	%	N = 4898	%			
Mortality (yes)	N = 364	114	6.71	250	5.1	1.33 (1.06 to 1.68)	6.2306	0.012
Mortality (no)	N = 6218	1570	93.29	4648	94.9			
Treatment failure (yes)	N = 94	26	2.03	70	2.29	0.88 (0.56 to 1.39)	0.2922	0.592
Treatment failure (no)	N = 6484	1658	98.5	4828	98.6			
Recurrence (yes)	N = 345	92	5.46	253	5.17	1.06 (0.83 to 1.35)	0.2238	0.636
Recurrence (no)	N = 6237	1592	94.54	4645	94.83			
Drug resistance (yes)	N = 444	135	8.02	309	6.31	1.29 (1.04 to 1.59)	5.811	0.016
Drug resistance (no)	N = 6138	1549	91.98	4589	93.69			

Table 2 shows the comparison of clinical outcomes between TB patients with HIV and those without HIV. 

**Table 2:** Comparison of clinical outcomes between tuberculosis (TB) patients with HIV and without HIV. * Chi-square test P value ≤ 0.05: significant

		With HIV		Without HIV		Odds ratio (CI)	Chi-square value	*p-value
		147	%	11158	%			
Mortality (yes)	N = 599	20	13.61	579	5.19	2.87 (1.78 to 4.64)	20.4817	<0.000
Mortality (no)	N = 10706	127	86.39	10579	94.81			
Treatment failure (yes)	N = 136	0	0	136	1.22	0.27 (0.01 to 4.45)	0.922	0.363
Treatment failure (no)	N = 11169	147	100	11022	98.78			
Recurrence (yes)	N = 574	7	5.04	567	5.1	0.98 (0.45 to 2.12)	0.0012	0.972
Recurrence (no)	N = 10731	140	94.96	10591	94.9			
Drug resistance (yes)	N = 594	12	8.16	582	5.22	1.64 (0.9 to 2.98)	2.6917	0.1
Drug resistance (no)	N = 10711	135	91.84	10576	94.78			

Patients with DM had 33% higher odds of dying from TB than those without DM (p=0.012). Additionally, individuals with DM were 29% more likely to develop drug resistance than those without (p=0.016). No significant relationship was found between treatment failure and drug resistance.

Patients with HIV were 87% more likely to die from TB than those without HIV (p<0.000). TB patients with HIV were 62% more likely to develop drug resistance to TB medications (p=0.1). Although TB patients with HIV had higher rates of treatment failure and recurrence, these relationships were not statistically significant (Table 2).

## Discussion

This study highlights significant trends in the rising prevalence of comorbidities, particularly DM and HIV, among TB patients in Tirunelveli district. The findings underscore the urgent need to address the dual burden of TB and comorbidities, which are increasingly recognized as critical factors influencing TB management and treatment outcomes.

Increasing burden of diabetes and TB

The rise in the proportion of TB patients with DM (from 3.5% in 2017 to 27% in 2021) reflects a growing challenge in managing TB in diabetic patients. Studies have shown that DM exacerbates the susceptibility to TB and complicates its treatment. The association between DM and TB is well-established, with individuals with DM being two to four times more likely to develop active TB due to compromised immunity and impaired granuloma formation [13]. The prevalence of DM among TB patients in India is rising, further complicating the efforts to control TB. Similar findings have been reported where the co-occurrence of TB and DM significantly impacts patient outcomes, including treatment failure and relapse [14].

The high prevalence of DM among TB patients in Pettai (25%) and Vadakkankulam (18%) highlights the geographical variation and underscores the need for region-specific interventions. Studies have shown that co-morbid diabetes increases the risk of MDR-TB and worsens prognosis, with a higher incidence of treatment failure [15].

The role of HIV in TB

The increasing prevalence of HIV among TB patients (from 0.7% in 2018 to 1.5% in 2021) is a critical concern. As HIV-positive individuals have a 20 times greater risk of developing TB compared to those without HIV [9], the rising number of TB-HIV co-infected patients exacerbates the burden on TB control programs. PLHIV face increased mortality and drug resistance rates due to delayed diagnosis and initiation of ART [16].

Impact on treatment outcomes

Patients with DM in this study had 33% higher odds of mortality, while HIV-positive TB patients had 87% higher odds, which aligns with previous studies indicating that both DM and HIV are associated with increased mortality and poor treatment outcomes in TB. For instance, a study found that TB patients with comorbidities had significantly higher mortality rates than those without comorbidities [17-19]. TB patients with comorbidities experience delayed treatment response, contributing to the worsening of clinical outcomes [20]. Similarly, the mortality rates were considerably higher among TB patients with untreated DM and delayed HIV treatment [21,22].

The risk of drug resistance among patients with DM (29%) and HIV (62%) reported in this study is concerning. In India, MDR-TB has been a growing problem, and comorbidities exacerbate this issue. It was demonstrated that the incidence of MDR-TB was significantly higher in TB patients with diabetes, as these individuals are less likely to adhere to the full course of treatment, increasing the risk of resistance [21,22]. Both DM and HIV have an even higher risk of developing drug resistance due to compromised immune responses and irregular treatment adherence [23].

Integrated care models

The findings suggest the need for integrated care that addresses both TB and its comorbidities, especially DM and HIV. Integrated care has been recognized as a critical approach in countries like India, where the dual burden of communicable and non-communicable diseases is significant. The importance of collaborative efforts between TB, diabetes, and HIV programs to improve diagnosis, treatment adherence, and patient outcomes [11]. The establishment of a comprehensive care model that includes regular screening for DM and HIV in all TB patients [17].

Integrated care models that combine TB treatment with diabetes management lead to better outcomes, including improved treatment adherence and reduced mortality [24]. Furthermore, early initiation of ART and anti-diabetic medications significantly reduced the risks of mortality and drug resistance among TB-HIV-DM co-infected patients.

Public health implications

The rising prevalence of both DM and HIV among TB patients necessitates urgent action to strengthen TB control efforts in India [13,14,16]. There is an immediate need to enhance surveillance systems for monitoring comorbidities, improve access to diagnostic services, and ensure better coordination between the TB, diabetes, and HIV control programs [13,14,21]. The scaling up of integrated screening programs to detect and manage comorbidities at the earliest stage possible, would improve outcomes and contribute to India's goal of eliminating TB by 2025 [22].

## Conclusions

This study highlights the evolving and significant challenge posed by the dual burden of TB and comorbidities, particularly DM and HIV, in Tirunelveli district, Tamil Nadu. The rising prevalence of these comorbidities among TB patients is a cause for concern, given the higher mortality rates and the increased risk of drug resistance among TB in patients with DM and HIV, it is crucial to strengthen collaboration between TB, diabetes, and HIV control programs. Efforts to improve patient adherence to treatment regimens, routine and timely diagnosis of comorbidities and linking of all to treatment, are essential for controlling the spread of TB and achieving better health outcomes. 
